# *tert*-Butyl *N*-(5-bromo-1*H*-imidazo[4,5-*b*]pyridin-2-ylmeth­yl)carbamate

**DOI:** 10.1107/S1600536808035393

**Published:** 2008-11-08

**Authors:** Ling Yin, Jiong Jia, Gui-Long Zhao, Jian-Wu Wang

**Affiliations:** a School of Chemistry and Chemical Engineering, Shandong University, Jinan 250100, People’s Republic of China; b The Pharmaceutical Research Institute of Tianjin, Tianjin 300193, People’s Republic of China

## Abstract

In the mol­ecule of the title compound, C_12_H_15_BrN_4_O_2_, the imidazole and pyridine rings are strictly coplanar [maximum deviation 0.006 (3) Å]. In the crystal structure, mol­ecules are linked into chains running parallel to the *a* axis by inter­molecular N—H⋯O hydrogen bonds. Centrosymmetrically related chains are further connected by N—H⋯N hydrogen-bonding inter­actions to form a two-dimensional layer structure parallel to the *ab* plane.

## Related literature

For general background on the properties of imidazole deriv­atives, see: Dai *et al.* (2004 ▶); Durant *et al.* (1973 ▶); Wang *et al.* (2007 ▶). For the crystal structures of related compounds, see: Lorenc *et al.* (2008 ▶).
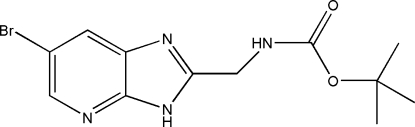


## Experimental

### 

#### Crystal data

C_12_H_15_BrN_4_O_2_
*M* *_r_* = 327.19
Orthorhombic, 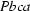

*a* = 10.7400 (11) Å
*b* = 9.6717 (9) Å
*c* = 28.215 (3) Å
*V* = 2930.8 (5) Å^3^
*Z* = 8
Mo *K*α radiation
μ = 2.81 mm^−1^
*T* = 298 (2) K
0.20 × 0.10 × 0.05 mm

#### Data collection

Bruker SMART APEX area-detector diffractometer
Absorption correction: multi-scan (*SADABS*; Bruker, 2002 ▶) *T*_min_ = 0.604, *T*_max_ = 0.872
16102 measured reflections
3374 independent reflections
2285 reflections with *I* > 2σ(*I*)
*R*_int_ = 0.031

#### Refinement

*R*[*F* ^2^ > 2σ(*F* ^2^)] = 0.038
*wR*(*F* ^2^) = 0.102
*S* = 1.01
3374 reflections
180 parameters
H atoms treated by a mixture of independent and constrained refinement
Δρ_max_ = 0.72 e Å^−3^
Δρ_min_ = −0.65 e Å^−3^

### 

Data collection: *SMART* (Bruker, 2002 ▶); cell refinement: *SAINT* (Bruker, 2002 ▶); data reduction: *SAINT*; program(s) used to solve structure: *SHELXS97* (Sheldrick, 2008 ▶); program(s) used to refine structure: *SHELXL97* (Sheldrick, 2008 ▶); molecular graphics: *ORTEP-3 for Windows* (Farrugia, 1997 ▶); software used to prepare material for publication: *SHELXL97*.

## Supplementary Material

Crystal structure: contains datablocks global, I. DOI: 10.1107/S1600536808035393/rz2259sup1.cif

Structure factors: contains datablocks I. DOI: 10.1107/S1600536808035393/rz2259Isup2.hkl

Additional supplementary materials:  crystallographic information; 3D view; checkCIF report

## Figures and Tables

**Table 1:** Hydrogen-bond geometry (Å, °)

*D*—H⋯*A*	*D*—H	H⋯*A*	*D*⋯*A*	*D*—H⋯*A*
N2—H2⋯N1^i^	0.81 (3)	2.12 (3)	2.911 (3)	165 (3)
N4—H3*A*⋯O1^ii^	0.84 (3)	1.98 (3)	2.822 (3)	178 (2)

Symmetry codes: (i) 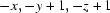; (ii) 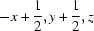.

**Fig. 1.**
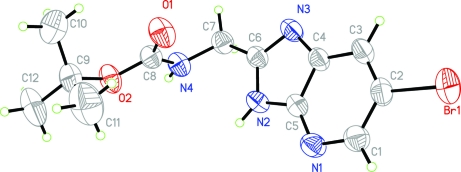
The molecular structure of the title compound with displacement ellipsoids drawn at the 30% probability level.

**Fig. 2.**
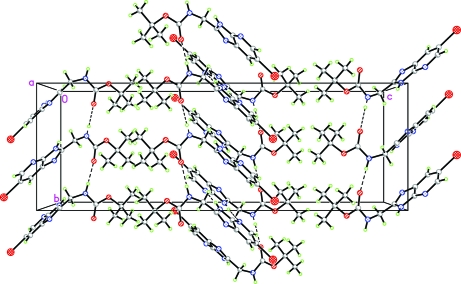
Crystal packing of the title compound viewed along the a axis. Intermolecular hydrogen bonds are shown as dashed lines.

## supplementary crystallographic information

### Comment

Nitrogen heterocyclic compounds and their derivatives are substances which show diverse biological activity (Dai *et al.*, 2004). Among them, imidazo[4,5-*b*]pyridine compounds are an important class of imidazole derivatives, which are widely used in the field of medicine (Durant *et al.*, 1973; Wang *et al.*, 2007). As a continuation of our studies on this subject, the structure of the title compound is described herein.

In the molecule of the title compound (Fig. 1) the imidazole and pyridine rings are strictly coplanar, the maximum deviation from the mean plane of the two rings being 0.006 (3) Å for atom C4. The C6—N2 and C6—N3 bond lengths in the imidazole ring are 1.362 (3) and 1.310 (3) Å, respectively; the bond angles between non-hydrogen atoms of the pyridine ring are in the range 114.3 (2)–126.8 (2)°, which is in line with the values reported for similar compounds (Lorenc *et al.*, 2008). In the crystal packing, intermolecular N—H···O hydrogen bonds involving the amide and carbonyl groups (Table 1) link adjacent molecules into chains parallel to the *a* axis. Centrosymmetrically related chains are further linked by intermolecular N—H···N hydrogen bonds to form a two-dimensional layer structure parallel to the *ab* plane (Fig. 2).

### Experimental

5-Bromopyridine-2,3-diamine(3.7 g, 20 mmol) and *N*-(*tert*-butoxycarbonyl)glycine (3.5 g, 20 mmol) were dissolved in THF (40 ml) and cooled to 273 K. *N*,*N*'-Dicyclohexylcarbodiimide (4.94 g, 24 mmol) was then added in batches and the mixture was stirred at 273 K for half an hour and at room temperature overnight. The filtrate was evaporated to afford a green solid, which was dissolved in acetic acid (20 ml) and the solution was stirred at 353 K for 8 h. The acetic acid was removed under reduced pressure and the crude title compound was separated as a pale green solid. Crystals of the title compound suitable for X-ray analysis were obtained by slow evaporation at room temperature if a dichloromethane-methanol (6:1, v/v) solution (yield; 70%, m.p. 475–476 K).

### Refinement

H atoms bound to N atoms were located from a difference Fourier map and refined freely. All other H atoms were placed at calculated positions and included in the refinement in the riding-model approximation, with C—H = 0.93-0.98 Å and *U*_iso_(H) = 1.2 *U*_eq_(C) or 1.5 *U*_eq_(C) for methylene and methyl H atoms.

### Crystal data

**Table:**

C_12_H_15_BrN_4_O_2_	*F*_000_ = 1328
*M**_r_* = 327.19	*D*_x_ = 1.483 Mg m^−^^3^
Orthorhombic, *P**b**c**a*	Mo *K*α radiation λ = 0.71073 Å
Hall symbol: -P 2ac 2ab	Cell parameters from 4297 reflections
*a* = 10.7400 (11) Å	θ = 2.4–24.9º
*b* = 9.6717 (9) Å	µ = 2.81 mm^−^^1^
*c* = 28.215 (3) Å	*T* = 298 (2) K
*V* = 2930.8 (5) Å^3^	Plate, colourless
*Z* = 8	0.20 × 0.10 × 0.05 mm

### Data collection

**Table:**

Bruker SMART APEX area-detector diffractometer	3374 independent reflections
Radiation source: fine-focus sealed tube	2285 reflections with *I* > 2σ(*I*)
Monochromator: graphite	*R*_int_ = 0.031
*T* = 298(2) K	θ_max_ = 27.6º
φ and ω scans	θ_min_ = 2.4º
Absorption correction: multi-scan(SADABS; Bruker, 2002)	*h* = −13→13
*T*_min_ = 0.604, *T*_max_ = 0.872	*k* = −12→8
16102 measured reflections	*l* = −36→29

### Refinement

**Table:**

Refinement on *F*^2^	Secondary atom site location: difference Fourier map
Least-squares matrix: full	Hydrogen site location: inferred from neighbouring sites
*R*[*F*^2^ > 2σ(*F*^2^)] = 0.038	H atoms treated by a mixture of independent and constrained refinement
*wR*(*F*^2^) = 0.102	*w* = 1/[σ^2^(*F*_o_^2^) + (0.0412*P*)^2^ + 2.0629*P*] where *P* = (*F*_o_^2^ + 2*F*_c_^2^)/3
*S* = 1.01	(Δ/σ)_max_ = 0.001
3374 reflections	Δρ_max_ = 0.72 e Å^−^^3^
180 parameters	Δρ_min_ = −0.65 e Å^−^^3^
Primary atom site location: structure-invariant direct methods	Extinction correction: none

### Special details

**Table:**

Geometry. All e.s.d.'s (except the e.s.d. in the dihedral angle between two l.s. planes) are estimated using the full covariance matrix. The cell e.s.d.'s are taken into account individually in the estimation of e.s.d.'s in distances, angles and torsion angles; correlations between e.s.d.'s in cell parameters are only used when they are defined by crystal symmetry. An approximate (isotropic) treatment of cell e.s.d.'s is used for estimating e.s.d.'s involving l.s. planes.
Refinement. Refinement of *F*^2^ against ALL reflections. The weighted *R*-factor *wR* and goodness of fit *S* are based on *F*^2^, conventional *R*-factors *R* are based on *F*, with *F* set to zero for negative *F*^2^. The threshold expression of *F*^2^ > σ(*F*^2^) is used only for calculating *R*-factors(gt) *etc*. and is not relevant to the choice of reflections for refinement. *R*-factors based on *F*^2^ are statistically about twice as large as those based on *F*, and *R*- factors based on ALL data will be even larger.

### Fractional atomic coordinates and isotropic or equivalent isotropic displacement parameters (Å^2^)

**Table:**

	*x*	*y*	*z*	*U*_iso_*/*U*_eq_	
Br1	0.13230 (4)	0.06577 (4)	0.356022 (12)	0.07448 (16)	
O1	0.2545 (2)	0.37496 (19)	0.61523 (7)	0.0588 (5)	
O2	0.1723 (2)	0.55519 (18)	0.65633 (7)	0.0532 (5)	
N1	0.0365 (2)	0.3710 (2)	0.45039 (8)	0.0492 (5)	
N2	0.1726 (2)	0.4846 (2)	0.50653 (8)	0.0425 (5)	
N3	0.35221 (19)	0.3775 (2)	0.48963 (8)	0.0481 (5)	
N4	0.2808 (2)	0.5942 (2)	0.59153 (8)	0.0441 (5)	
C1	0.0386 (3)	0.2736 (3)	0.41650 (10)	0.0508 (7)	
H1	−0.0350	0.2529	0.4006	0.061*	
C2	0.1457 (3)	0.2029 (3)	0.40420 (9)	0.0488 (6)	
C3	0.2584 (3)	0.2271 (3)	0.42539 (9)	0.0499 (6)	
H3	0.3301	0.1795	0.4167	0.060*	
C4	0.2590 (2)	0.3268 (3)	0.46047 (8)	0.0426 (6)	
C5	0.1455 (2)	0.3930 (3)	0.47101 (9)	0.0395 (5)	
C6	0.2964 (2)	0.4704 (3)	0.51590 (9)	0.0414 (6)	
C7	0.3610 (2)	0.5565 (3)	0.55229 (10)	0.0463 (6)	
H7A	0.4320	0.5056	0.5644	0.056*	
H7B	0.3920	0.6400	0.5374	0.056*	
C8	0.2370 (2)	0.4976 (3)	0.62088 (9)	0.0411 (6)	
C9	0.1098 (3)	0.4696 (3)	0.69213 (10)	0.0563 (7)	
C10	0.2031 (4)	0.3846 (5)	0.71874 (13)	0.0989 (13)	
H10A	0.2389	0.3172	0.6978	0.148*	
H10B	0.1625	0.3384	0.7446	0.148*	
H10C	0.2675	0.4436	0.7308	0.148*	
C11	0.0119 (4)	0.3815 (5)	0.66859 (16)	0.1005 (14)	
H11A	0.0515	0.3131	0.6491	0.151*	
H11B	−0.0406	0.4389	0.6493	0.151*	
H11C	−0.0375	0.3366	0.6924	0.151*	
C12	0.0497 (4)	0.5769 (4)	0.72365 (14)	0.0929 (13)	
H12A	0.1132	0.6315	0.7386	0.139*	
H12B	0.0005	0.5317	0.7474	0.139*	
H12C	−0.0027	0.6358	0.7049	0.139*	
H2	0.124 (3)	0.537 (3)	0.5189 (11)	0.051 (9)*	
H3A	0.272 (2)	0.678 (3)	0.5990 (8)	0.034 (7)*	

### Atomic displacement parameters (Å^2^)

**Table:**

	*U*^11^	*U*^22^	*U*^33^	*U*^12^	*U*^13^	*U*^23^
Br1	0.0908 (3)	0.0764 (3)	0.0563 (2)	0.00984 (19)	0.00153 (17)	−0.02719 (16)
O1	0.0800 (14)	0.0302 (10)	0.0660 (12)	0.0025 (9)	0.0186 (11)	−0.0051 (9)
O2	0.0700 (13)	0.0381 (10)	0.0516 (11)	0.0002 (9)	0.0197 (9)	−0.0059 (8)
N1	0.0436 (12)	0.0482 (13)	0.0559 (14)	0.0046 (10)	−0.0002 (10)	−0.0114 (11)
N2	0.0382 (12)	0.0406 (12)	0.0488 (13)	0.0050 (10)	0.0065 (10)	−0.0055 (10)
N3	0.0387 (11)	0.0575 (14)	0.0482 (12)	0.0069 (10)	0.0078 (10)	−0.0079 (11)
N4	0.0540 (14)	0.0259 (11)	0.0524 (13)	−0.0004 (9)	0.0092 (10)	−0.0055 (9)
C1	0.0515 (16)	0.0472 (16)	0.0539 (16)	0.0033 (13)	−0.0026 (13)	−0.0078 (13)
C2	0.0593 (17)	0.0493 (15)	0.0379 (13)	0.0037 (13)	0.0066 (12)	−0.0044 (11)
C3	0.0513 (16)	0.0556 (16)	0.0427 (14)	0.0107 (13)	0.0110 (12)	−0.0047 (12)
C4	0.0414 (13)	0.0478 (15)	0.0385 (12)	0.0051 (11)	0.0104 (11)	0.0005 (11)
C5	0.0417 (13)	0.0368 (13)	0.0401 (13)	0.0018 (11)	0.0069 (11)	−0.0008 (10)
C6	0.0391 (13)	0.0430 (14)	0.0420 (14)	−0.0003 (11)	0.0083 (11)	0.0012 (11)
C7	0.0411 (13)	0.0492 (15)	0.0486 (15)	−0.0048 (12)	0.0061 (12)	−0.0017 (12)
C8	0.0457 (14)	0.0332 (13)	0.0445 (13)	−0.0005 (11)	0.0024 (11)	−0.0051 (11)
C9	0.0630 (18)	0.0561 (17)	0.0499 (16)	−0.0051 (14)	0.0154 (14)	−0.0002 (13)
C10	0.109 (3)	0.124 (3)	0.064 (2)	0.021 (3)	0.009 (2)	0.025 (2)
C11	0.085 (3)	0.115 (3)	0.101 (3)	−0.044 (3)	0.024 (2)	−0.015 (3)
C12	0.109 (3)	0.093 (3)	0.077 (2)	0.001 (2)	0.045 (2)	−0.012 (2)

### Geometric parameters (Å, °)

**Table:**

Br1—C2	1.904 (3)	C3—H3	0.9300
O1—C8	1.211 (3)	C4—C5	1.409 (3)
O2—C8	1.339 (3)	C6—C7	1.493 (4)
O2—C9	1.469 (3)	C7—H7A	0.9700
N1—C5	1.325 (3)	C7—H7B	0.9700
N1—C1	1.342 (3)	C9—C10	1.498 (5)
N2—C6	1.362 (3)	C9—C11	1.507 (5)
N2—C5	1.369 (3)	C9—C12	1.512 (4)
N2—H2	0.81 (3)	C10—H10A	0.9600
N3—C6	1.310 (3)	C10—H10B	0.9600
N3—C4	1.385 (3)	C10—H10C	0.9600
N4—C8	1.335 (3)	C11—H11A	0.9600
N4—C7	1.449 (3)	C11—H11B	0.9600
N4—H3A	0.84 (3)	C11—H11C	0.9600
C1—C2	1.383 (4)	C12—H12A	0.9600
C1—H1	0.9300	C12—H12B	0.9600
C2—C3	1.370 (4)	C12—H12C	0.9600
C3—C4	1.382 (3)		
			
C8—O2—C9	121.1 (2)	N4—C7—H7B	109.0
C5—N1—C1	114.3 (2)	C6—C7—H7B	109.0
C6—N2—C5	106.5 (2)	H7A—C7—H7B	107.8
C6—N2—H2	128 (2)	O1—C8—N4	123.3 (2)
C5—N2—H2	126 (2)	O1—C8—O2	125.9 (2)
C6—N3—C4	104.4 (2)	N4—C8—O2	110.8 (2)
C8—N4—C7	120.4 (2)	O2—C9—C10	110.4 (3)
C8—N4—H3A	118.5 (17)	O2—C9—C11	109.5 (3)
C7—N4—H3A	120.2 (17)	C10—C9—C11	112.1 (3)
N1—C1—C2	122.6 (3)	O2—C9—C12	102.3 (2)
N1—C1—H1	118.7	C10—C9—C12	111.6 (3)
C2—C1—H1	118.7	C11—C9—C12	110.5 (3)
C3—C2—C1	122.8 (2)	C9—C10—H10A	109.5
C3—C2—Br1	119.8 (2)	C9—C10—H10B	109.5
C1—C2—Br1	117.4 (2)	H10A—C10—H10B	109.5
C2—C3—C4	115.9 (2)	C9—C10—H10C	109.5
C2—C3—H3	122.1	H10A—C10—H10C	109.5
C4—C3—H3	122.1	H10B—C10—H10C	109.5
C3—C4—N3	132.5 (2)	C9—C11—H11A	109.5
C3—C4—C5	117.7 (2)	C9—C11—H11B	109.5
N3—C4—C5	109.8 (2)	H11A—C11—H11B	109.5
N1—C5—N2	127.9 (2)	C9—C11—H11C	109.5
N1—C5—C4	126.8 (2)	H11A—C11—H11C	109.5
N2—C5—C4	105.4 (2)	H11B—C11—H11C	109.5
N3—C6—N2	114.0 (2)	C9—C12—H12A	109.5
N3—C6—C7	124.0 (2)	C9—C12—H12B	109.5
N2—C6—C7	122.1 (2)	H12A—C12—H12B	109.5
N4—C7—C6	113.0 (2)	C9—C12—H12C	109.5
N4—C7—H7A	109.0	H12A—C12—H12C	109.5
C6—C7—H7A	109.0	H12B—C12—H12C	109.5
			
C5—N1—C1—C2	−0.2 (4)	N3—C4—C5—N2	0.5 (3)
N1—C1—C2—C3	−0.4 (4)	C4—N3—C6—N2	0.4 (3)
N1—C1—C2—Br1	179.1 (2)	C4—N3—C6—C7	−178.3 (2)
C1—C2—C3—C4	0.5 (4)	C5—N2—C6—N3	−0.1 (3)
Br1—C2—C3—C4	−178.99 (19)	C5—N2—C6—C7	178.7 (2)
C2—C3—C4—N3	179.1 (3)	C8—N4—C7—C6	65.8 (3)
C2—C3—C4—C5	0.0 (4)	N3—C6—C7—N4	−149.7 (2)
C6—N3—C4—C3	−179.6 (3)	N2—C6—C7—N4	31.7 (3)
C6—N3—C4—C5	−0.5 (3)	C7—N4—C8—O1	−4.5 (4)
C1—N1—C5—N2	−179.8 (3)	C7—N4—C8—O2	175.7 (2)
C1—N1—C5—C4	0.7 (4)	C9—O2—C8—O1	−2.6 (4)
C6—N2—C5—N1	−179.8 (3)	C9—O2—C8—N4	177.2 (2)
C6—N2—C5—C4	−0.3 (3)	C8—O2—C9—C10	61.7 (4)
C3—C4—C5—N1	−0.7 (4)	C8—O2—C9—C11	−62.3 (4)
N3—C4—C5—N1	−179.9 (2)	C8—O2—C9—C12	−179.5 (3)
C3—C4—C5—N2	179.8 (2)		

### Hydrogen-bond geometry (Å, °)

**Table:**

*D*—H···*A*	*D*—H	H···*A*	*D*···*A*	*D*—H···*A*
N2—H2···N1^i^	0.81 (3)	2.12 (3)	2.911 (3)	165 (3)
N4—H3A···O1^ii^	0.84 (3)	1.98 (3)	2.822 (3)	178 (2)

Symmetry codes: (i) −*x*, −*y*+1, −*z*+1; (ii) −*x*+1/2, *y*+1/2, *z*.
